# Supplementary material for: Pathology and Immunity After SARS-CoV-2 Infection in Male Ferrets Is Affected by Age and Inoculation Route
Source: Front Immunol. 2021 Oct 21;12:750229. doi: 10.3389/fimmu.2021.750229 (PMC8566349; doi:10.3389/fimmu.2021.750229)
Supplement: Supplementary file 1 [file DataSheet_1.pdf]

Supplemental materials

Pathology and immunity after SARS-CoV-2 infection in male ferrets is affected by age and inoculation route

Koen van de Ven[1], Harry van Dijken[1], Lisa Wijsman[1], Angéla Gomersbach[2], Tanja Schouten[2], Jolanda Kool[1], Stefanie Lenz[1], Paul Roholl[3], Adam Meijer[1], Puck B. van Kasteren[1], Jørgen de Jonge[1#]

[1]Centre for Infectious Disease Control, National Institute for Public Health and the Environment (RIVM), Bilthoven, the Netherlands
[2]Animal Research Centre, Poonawalla Science Park, Bilthoven, The Netherlands
[3]Microscope Consultancy, Weesp, the Netherlands
[#]Correspondence should be addressed to jorgen.de.jonge@rivm.nl

Supplemental Table 1 | Immune status before SARS-CoV-2 infection

Ferret	Age	Inocu-lation route	Section day post infection	HI (dilution)			ELISA (titer)				
				(B/ Colorado /06/2017)	(B/ Maryland/ 15/2016)	(A/ Michigan/ 45/15)	Aleution IgG	CDV[1]	FCOV[2]	CCoV[3]	NL-63
1	Young	i.n.	5	neg	neg	neg	<100	280	109	<100	<100
2	Young	i.n.	5	neg	neg	neg	<100	77	<100	<100	<100
3	Young	i.n.	5	neg	neg	neg	<100	360	<100	<100	<100
4	Young	i.n.	14	neg	neg	neg	<100	224	<100	<100	<100
5	Young	i.n.	14	neg	neg	neg	<100	331	126	<100	<100
6	Young	i.n.	14	neg	neg	neg	<100	76	<100	<100	<100
7	Young	i.n.	21	neg	neg	neg	<100	984	<100	450	410
8	Young	i.n.	21	neg	neg	neg	<100	1058	142	163	111
9	Young	i.n.	21	neg	neg	neg	<100	1050	136	506	239
10	Young	i.t.	5	neg	neg	neg	<100	675	<100	<100	<100
11	Young	i.t.	5	neg	neg	neg	<100	570	<100	107	<100
12	Young	i.t.	5	neg	neg	neg	<100	319	<100	<100	<100
13	Young	i.t.	14	neg	neg	neg	<100	267	160	<100	<100
14	Young	i.t.	14	neg	neg	neg	<100	388	106	145	<100
15	Young	i.t.	14	neg	neg	neg	<100	54	150	<100	<100
16	Young	i.t.	21	neg	neg	neg	<100	1053	<100	301	354
17	Young	i.t.	21	neg	neg	neg	<100	1058	123	183	170
18	Young	i.t.	21	neg	neg	neg	<100	1054	121	517	373
19	Adult	i.n.	5	neg	neg	neg	<100	160	134	<100	<100
20	Adult	i.n.	5	neg	neg	neg	<100	102	<100	<100	<100
21	Adult	i.n.	5	neg	neg	neg	<100	213	<100	<100	<100
22	Adult	i.t.	21	neg	neg	neg	<100	505	142	105	<100
23	Adult	i.t.	21	neg	neg	neg	<100	215	113	<100	<100
24	Adult	i.t.	21	neg	neg	neg	<100	675	<100	197	<100
25	Adult	i.t.	5	neg	neg	neg	<100	347	172	<100	<100
26	Adult	i.t.	5	neg	neg	neg	<100	504	116	<100	<100
27	Adult	i.t.	5	neg	neg	neg	<100	445	174	122	<100
28	Adult	i.t.	14	neg	neg	neg	<100	511	<100	158	<100
29	Adult	i.t.	14	neg	neg	neg	<100	206	<100	<100	<100
31	Adult	i.n.	21	neg	neg	neg	115	125	140	<100	<100
32	Adult	i.n.	21	neg	neg	neg	<100	312	<100	<100	<100
33	Adult	i.n.	21	neg	neg	neg	<100	669	<100	189	<100
34	Young	Mock	14	neg	neg	neg	<100	12	<100	<100	<100
35	Young	Mock	14	neg	neg	neg	149	469	424	116	<100
36	Young	Mock	14	neg	neg	neg	100	276	138	<100	<100
37	Adult	Mock	21	neg	neg	>320	<100	278	<100	<100	<100
38	Adult	Mock	21	neg	neg	160	<100	404	114	107	<100
39	Adult	Mock	21	neg	neg	>320	<100	28	<100	<100	<100

[1]CDV = Canine distemper virus
[2]FCOV = Feline coronavirus, representative of ferret systemic corona virus
[3]CCoV = Canine coronavirus, representative of ferret enteric corona virus

Pre-existing immunity against CCoV and NL-63, but not SARS-CoV-2
Pre-existing cellular responses against SARS-CoV-2
Pre-existing humoral responses against SARS-CoV-2.

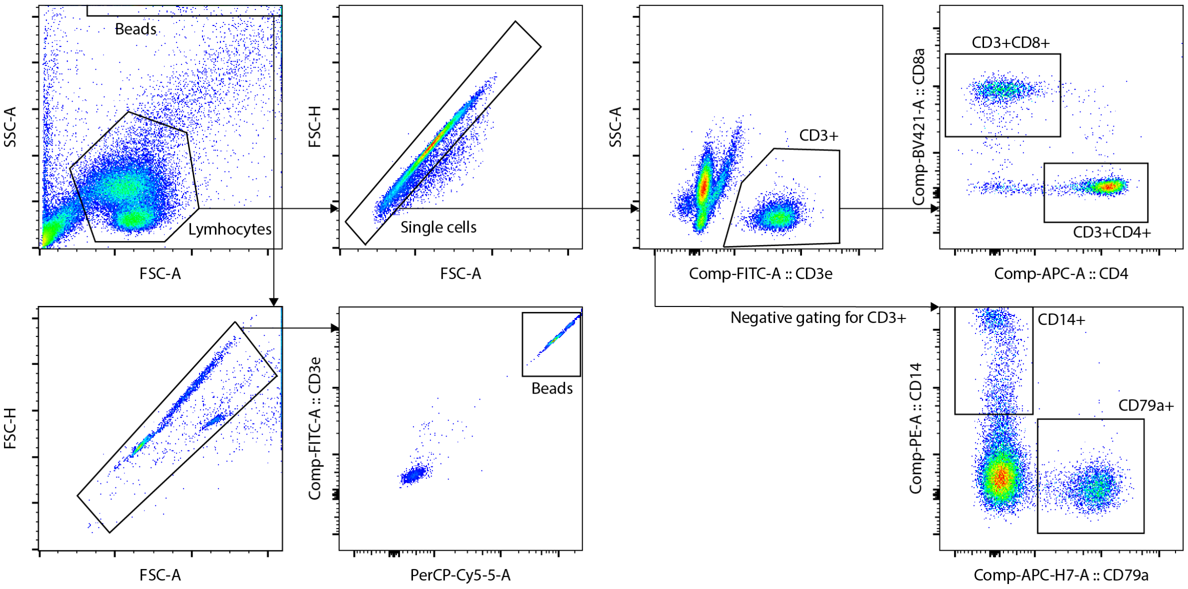

Supplemental figure 1: Gating strategy of whole blood trucount. Plots show the gating strategy for identification of cell subsets in whole blood of young and adult ferrets. CD14 and CD79a subsets were gated in the 'Single cells' population excluding all CD3+ cells. Beads were used to correct cell counts for the volume of measured blood.

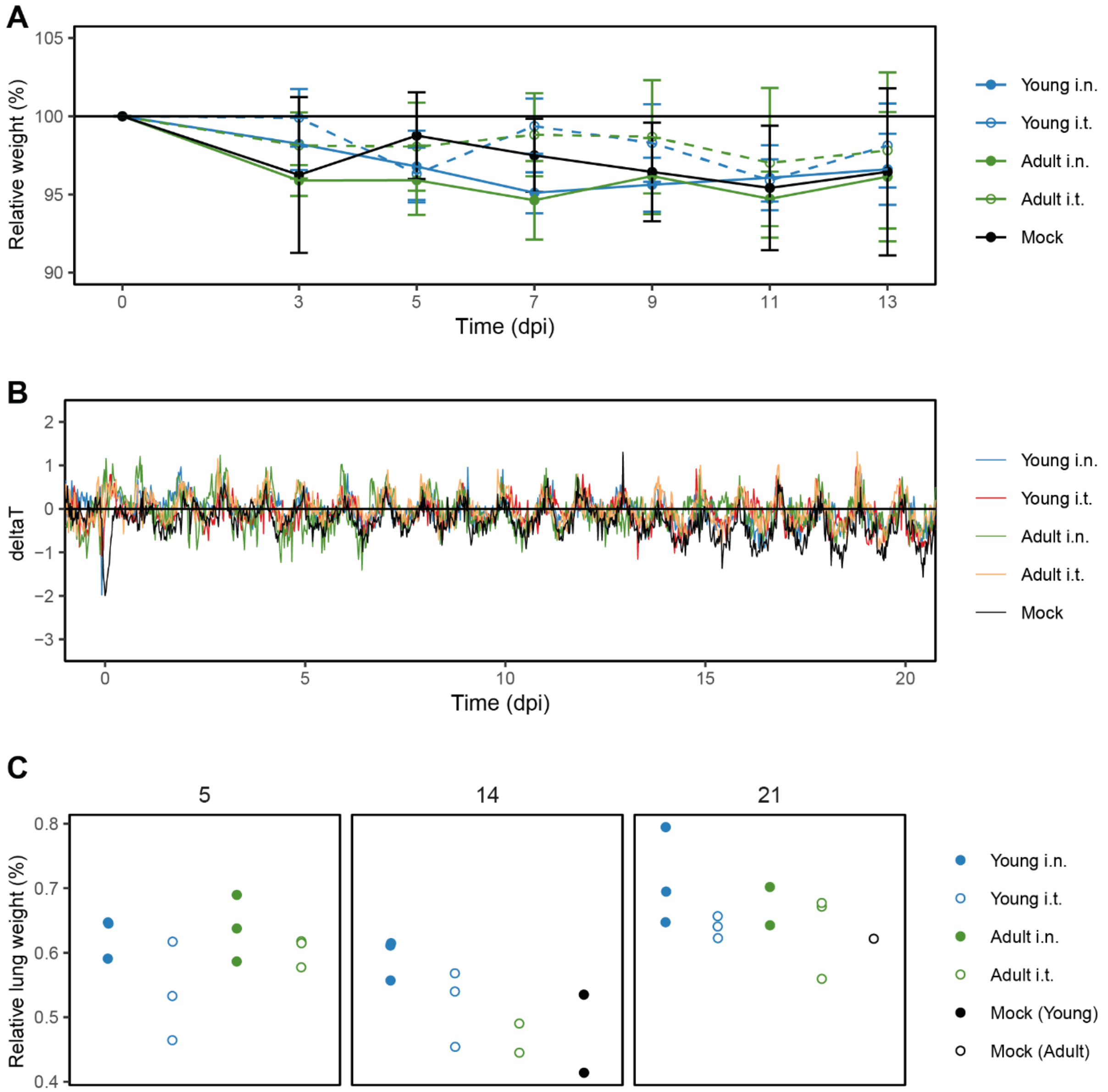

Supplemental figure 2: SARS-CoV-2 infection does not induce clinical disease in male ferrets. A) Body weight was measured on various time points and depicted as % of original bodyweight on the day of infection. **B)** Body temperature was measured continuously in 30-minute intervals by implanted abdominal transponders. The ΔT was calculated by subtracting body temperature during baseline (1-6 days before infection) from the body temperature after SARS-CoV-2 infection. **C)** Relative lung weight depicted as a percentage of total body weight on the day of infection. The different panels depict the relative lung weight on 5, 14 and 21 days post infection (dpi). Lines **(A, B)** depict the group mean while the error bars depict standard error of the mean (**A** only). For **(A, B)**: n = 3-6; for **(C)**: with exception of ‘Adult i.t.’ on 14 dpi (n = 2), all groups are n = 3.

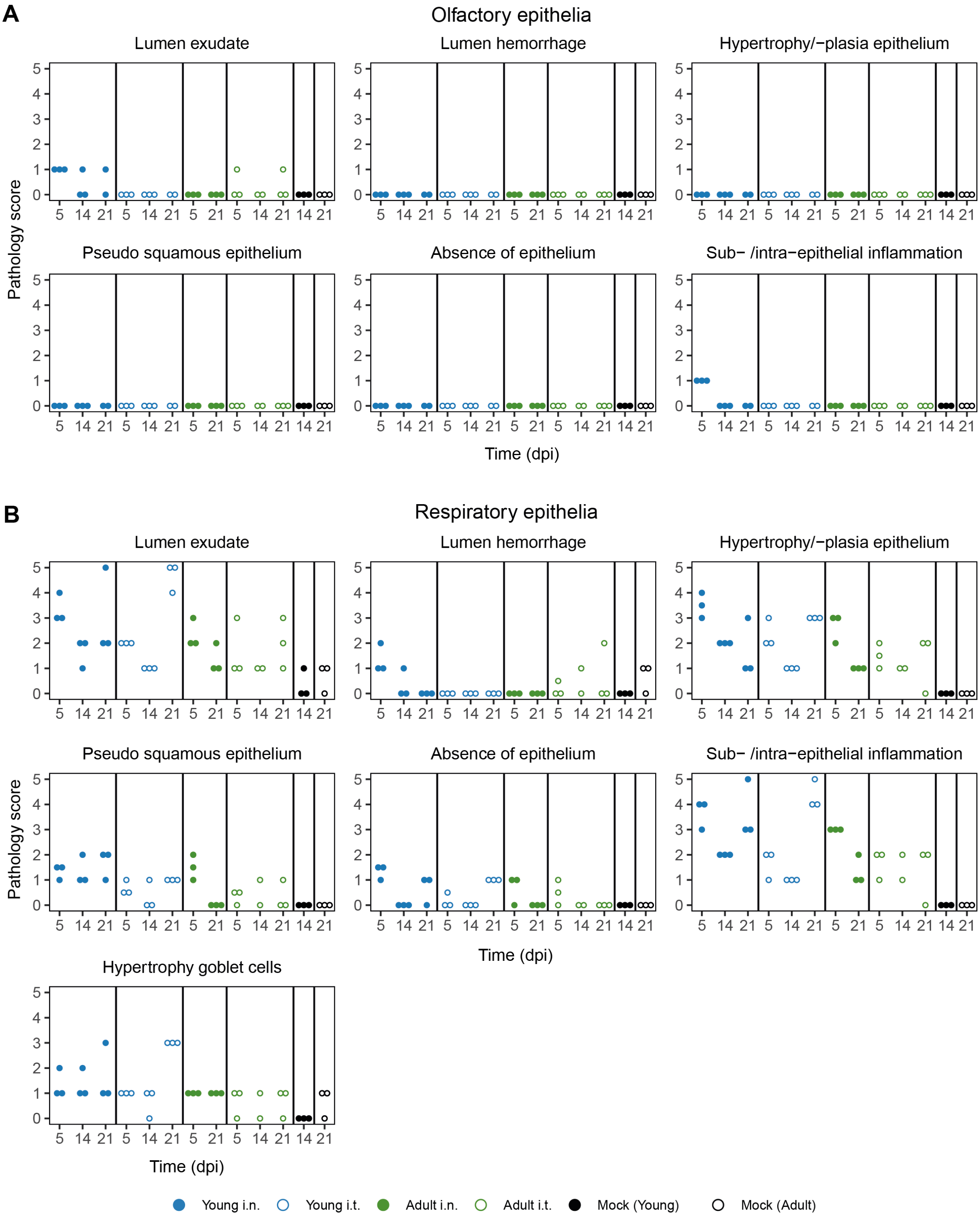

Supplemental figure 3: Extensive pathology scoring nasal turbinates. A, B) Scoring of **(A)** olfactory and **(B)** respiratory turbinates. Panels depict individual parameters related to epithelial damage and inflammation on 5, 14 and 21 days post infection (dpi). The infection-induced pathology was scored on a scale of 0–5 based on the parameters described in the materials and methods. With exception of 'Adult i.t.' on 14 dpi (n = 2), all groups are n = 3.

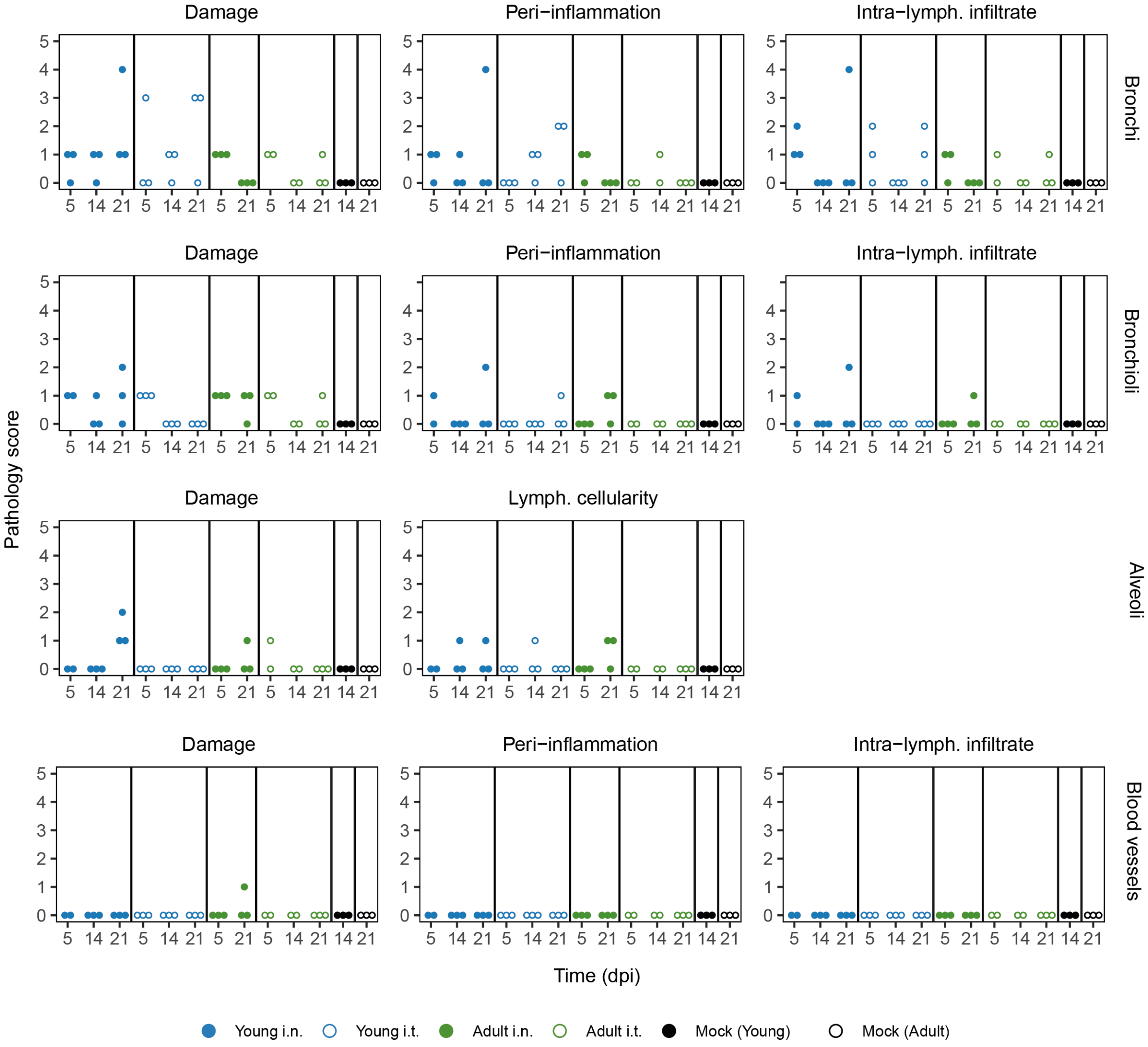

Supplemental figure 4: Extensive pathology scoring lungs. Panels show individual scoring by parameters related to epithelial damage and inflammation. The infection-induced pathology was scored on a scale of 0–5 based on the parameters described in the materials and methods on 5, 14 and 21 days post infection (dpi). With exception of ‘Adult i.t.’ on 14 dpi (n = 2), all groups are n = 3.

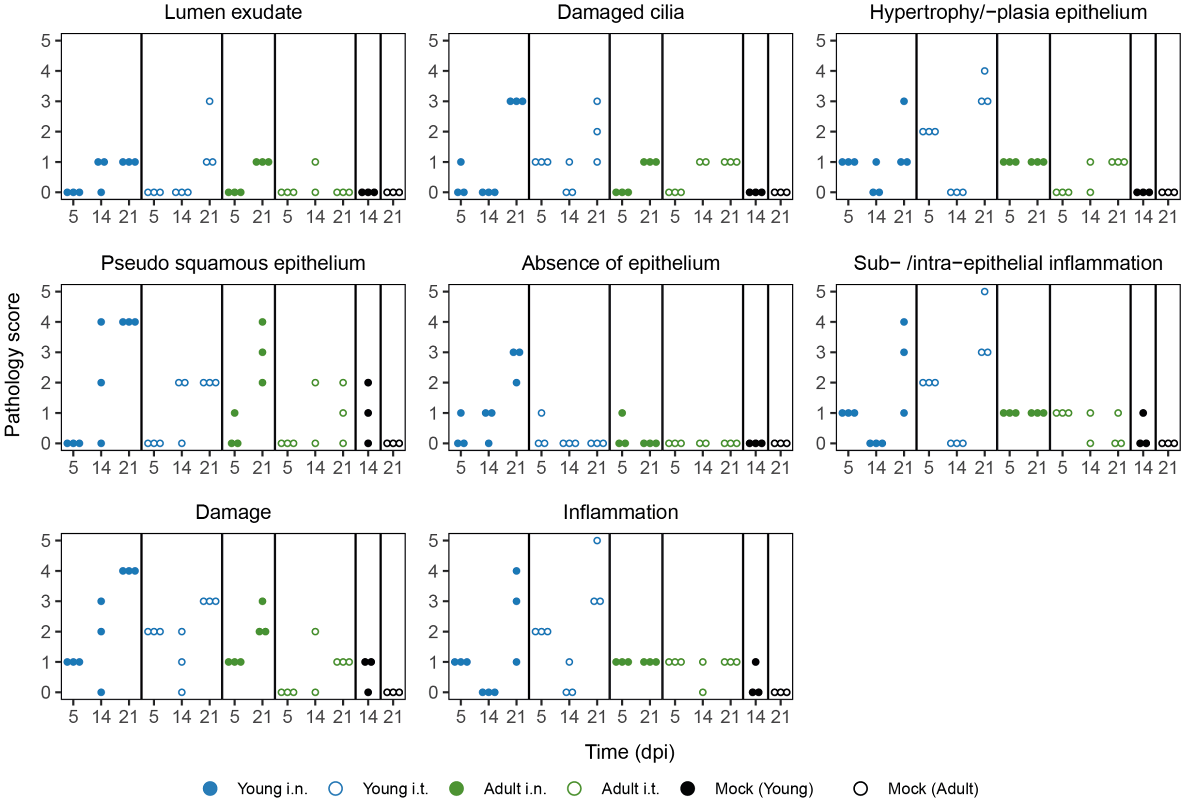

Supplemental figure 5: Extensive pathology scoring trachea. Panels show individual scoring by parameters related to epithelial damage and inflammation. The infection-induced pathology was scored on a scale of 0–5 based on the parameters described in the materials and methods on 5, 14 and 21 days post infection (dpi). With exception of 'Adult i.t.' on 14 dpi (n = 2), all groups are n = 3.

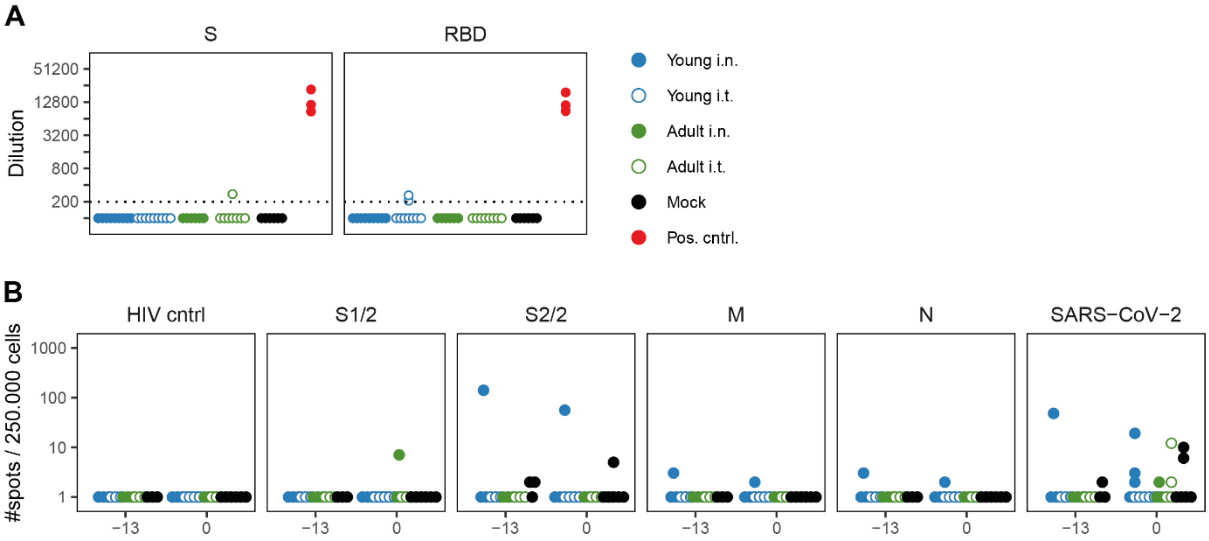

Supplemental figure 6: Pre-existing immune responses against SARS-CoV-2 in ferrets. **A)** Sera from three animals before SARS-CoV-2 infection contained small antibody responses against (the receptor binding domain [RBD] of) spike (S) in ELISA assays, but these were different animals from the one that responded in the ELISpot of panel B. Responses are depicted as the (modelled) dilution at which the ELISA curve drops below background (mean + 3x SD of SARS-CoV-2 naïve animals at 200x dilution). The dotted line indicates the lowest dilution tested and negative samples were set to half that dilution for visualization purposes. Positive control consists of sera of SARS-CoV-2 infected animals collected 21 days post infection (dpi). N= 3 for positive control sera and n = 6-9 for other groups. **B)** IFNγ-ELISpots performed with PBMCs isolated 13 and 0 days before SARS-CoV-2 infection indicate that one animal already possessed (cross-reactive) T cell responses against overlapping peptide pools of the S-protein of SARS-CoV-2. Data shown were corrected for medium background and were set to a minimum of 1 spot for visualization on a log-scale. N = 3 at -13 dpi and n = 5-6 for 0 dpi.

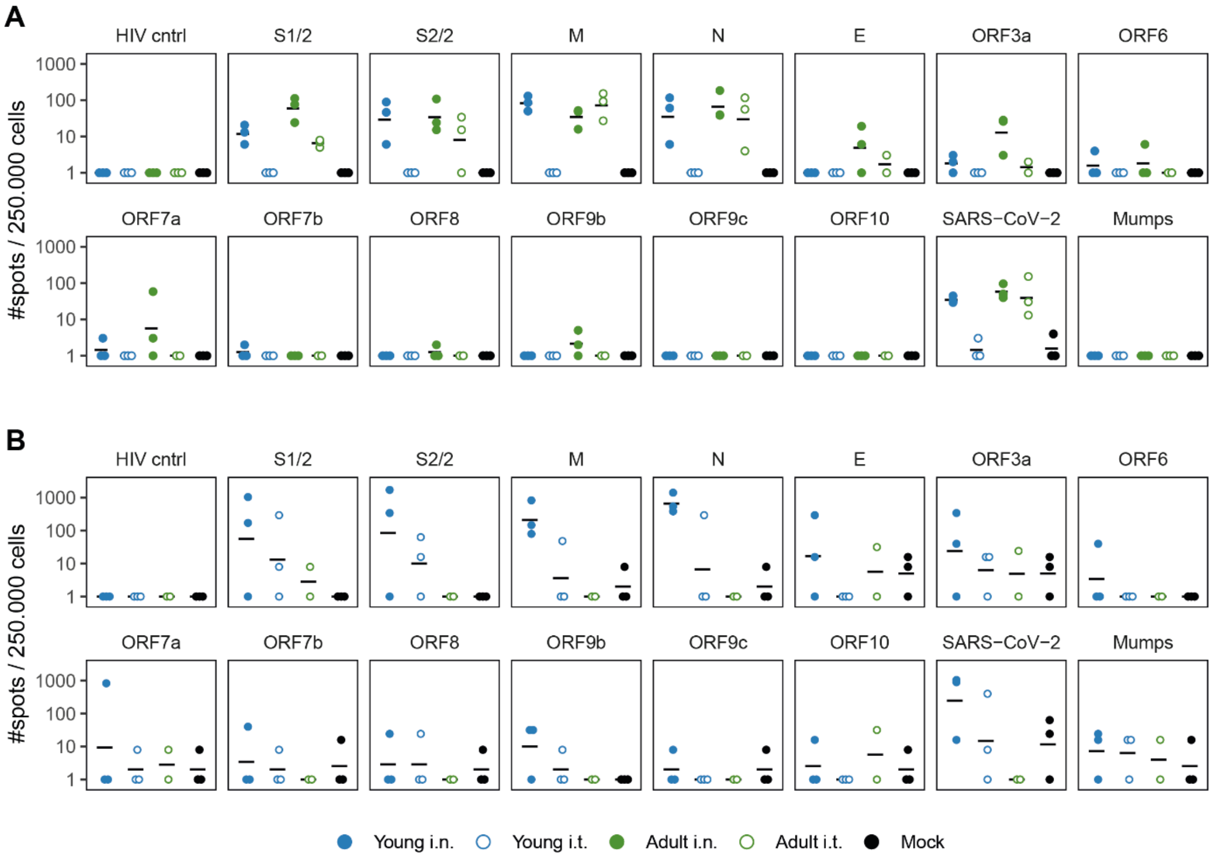

Supplemental figure 7: Cellular responses against SARS-CoV-2 in PBMC and lung. A, B) Cellular responses in PBMCs **(A)** and lung derived lymphocytes **(B)** as determined by IFNγ-ELISpot. Cells were stimulated with various SARS-CoV-2 peptide pools or live virus. Dots show individual ferrets while black lines indicate the group geometric mean. Data were corrected for medium background and were set to a minimum of 1 spot for visualization on a log-scale. **A)** Responses in PBMC isolated 21 days post infection (dpi). **B)** Responses of lung-derived lymphocytes 14 dpi. N = 3 for all panels, with exception of 'Adult i.t.' on 14 dpi (n = 2).
